# Functional Mandibular Reconstruction With Integrated Nerve Repair: A Pathway to Dental Implant Rehabilitation

**DOI:** 10.7759/cureus.89533

**Published:** 2025-08-07

**Authors:** Katsumi Shinozaki, Shogo Kikuta, Satoshi Moriguchi, Keita Todoroki, Jingo Kusukawa

**Affiliations:** 1 Dental and Oral Medical Center, Kurume University School of Medicine, Kurume, JPN; 2 Dental and Oral Medical Center, Kurume University School of Medicine, kurume, JPN

**Keywords:** dental implants, mandibular nerve, mandibular reconstruction, nerve conduit, nerve reconstruction

## Abstract

Functional reconstruction of large mandibular defects, especially in young patients, presents a significant clinical challenge. The ideal approach should not only restore skeletal contour but also address nerve deficits and facilitate final occlusal rehabilitation, all while minimizing morbidity. This report describes a comprehensive, multi-staged strategy for such a case.

A 22-year-old male patient was diagnosed with a large cemento-ossifying fibroma (COF) located in the molar region of the left mandible. He underwent a segmental mandibulectomy, resulting in a 40-mm defect. Immediate reconstruction was performed using a custom-made titanium mesh tray filled with autologous particulate cancellous bone and marrow (PCBM). Concurrently, the 40-mm gap in the inferior alveolar nerve (IAN) was bridged with a polyglycolic acid (PGA)-collagen nerve conduit. Subsequent procedures included secondary bone augmentation at 18 months and placement of two dental implants at 30 months, followed by final prosthetic delivery. At the 6.5-year follow-up, the patient showed excellent bone stability with no tumor recurrence and was highly satisfied with both the functional and aesthetic outcomes. Postoperative imaging confirmed successful graft integration and the formation of a new canal-like structure. While quantitative sensory testing indicated only partial nerve recovery, this was attributed to the large 40-mm defect size, which was at the upper limit of the conduit's indication. This case demonstrates that a holistic approach, integrating custom-made devices for both skeletal and neural repair, provides a viable pathway to full functional rehabilitation and represents a valuable treatment paradigm for improving quality of life in young patients with extensive mandibular defects.

## Introduction

Mandibular reconstruction following segmental mandibulectomy due to tumors, osteomyelitis, or osteoradionecrosis is essential for the restoration of facial aesthetics and vital oral functions, including mastication and speech [1-5]. While vascularized free flaps are often considered the standard of care for extensive defects, this approach presents significant challenges, notably donor site morbidity and prolonged surgical durations [1-6]. These considerations are particularly critical in patients diagnosed with benign pathologies, for whom minimizing surgical invasiveness while preserving long-term quality of life is a primary objective [5,6].

Recent advances in three-dimensional (3D) printing technology have established patient-specific titanium implants as a viable alternative for mandibular reconstruction [1,2,4,6]. Fabricated to precisely match the dimensions and morphology of the defect, these implants ensure high fidelity of fit, shorten operative duration, and enable a more accurate restoration of mandibular contours [1,2,4,6]. Nevertheless, significant challenges persist in achieving comprehensive functional restoration, particularly concerning post-operative neurosensory deficits and occlusal rehabilitation following segmental mandibulectomy.

The present case report describes the comprehensive reconstruction of a large mandibular defect following resection of a cemento-ossifying fibroma (COF) in an adult male patient. Our integrated approach combined skeletal reconstruction, using a custom-made titanium mesh tray with a particulate cancellous bone and marrow (PCBM) graft, and simultaneous inferior alveolar nerve (IAN) repair with a polyglycolic acid (PGA)-collagen conduit. This strategy ultimately facilitated a multi-staged prosthetic rehabilitation with dental implants, restoring long-term function and aesthetics.

## Case presentation

**Figure 1:**
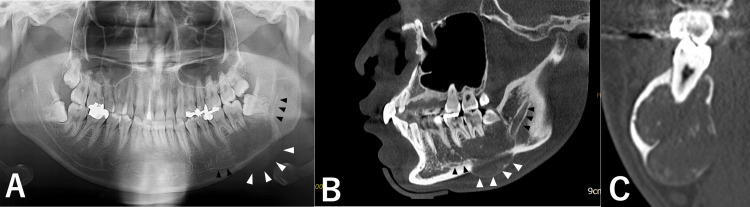
Panoramic and CBCT findings A: Panoramic image. Panoramic radiograph shows an expansile lesion extending beyond the inferior border of the mandible (white arrowheads), with inferior displacement of the mandibular canal (black arrowheads). B, C: CBCT image. CBCT shows expansile lesion with mixed densities and partially ill-defined borders, extending beyond the mandibular border (white arrowheads), displacing the mandibular canal inferiorly (black arrowheads), and partially encasing the neurovascular bundle (C). CBCT: Cone-beam CT

A 22-year-old male patient presented to our hospital (Dental and Oral Medical Center, Kurume University School of Medicine, Kurume, Japan) with a chief complaint of painless swelling in the left mandible. He presented with a one-year history of a progressive, asymptomatic expansion of the left mandible. He had no significant medical or family history and no history of smoking or alcohol consumption. Clinical examination revealed noticeable facial asymmetry corresponding to a hard, diffuse, and bony swelling in the left posterior mandible, with no associated neurosensory deficits. Panoramic radiography showed a lesion causing root resorption of adjacent teeth (Figure 1A). Cone-beam CT (CBCT) further characterized this as anexpansile lesion with internal mixed densities and partially ill-defined margins, extending beyond the inferior border of the mandible and causing inferior displacement of the mandibular canal (Figure 1B), as well as partial encasement and obscuration of the neurovascular bundle(Figure 1C).

Subsequent MRI suggested a significant fibrous component. In January 2019, an incisional biopsy confirmed the diagnosis of COF.

**Figure 2:**
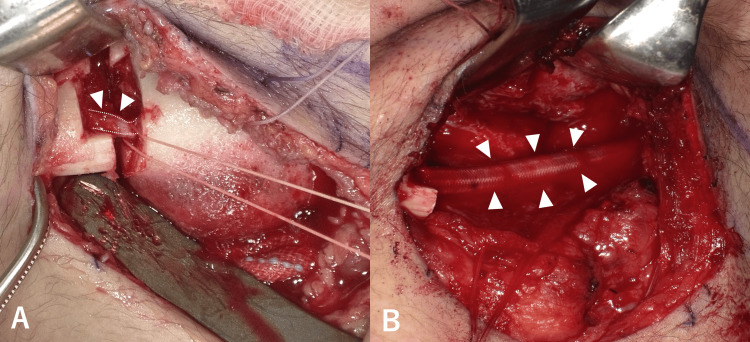
Intraoperative findings A: Segmental mandibulectomy. IAN in the center is seen (arrowheads). B: Reconstruction of IAN using a PGA-collagen nerve conduit (arrowheads). IAN: Inferior alveolar nerve; PGA: Polyglycolic acid

In March 2019, the patient underwent a segmental mandibulectomy, resecting the segment containing teeth 36 through 38 (Figure 2A). This created a 40-mm skeletal and nerve defect, which was addressed with immediate functional reconstruction. The mandibular contour was restored using a custom-made titanium mesh tray (Ultra Flex Mesh Custom®, Okada Medical Materials, Japan), which had been pre-shaped on a 3D-printed anatomical model. Concurrently, the 40-mm gap in the IAN was bridged using a 4.0-mm diameter PGA-collagen nerve conduit (Nerve Bridge®, Toyobo, Japan) (Figure 2B).

**Figure 3:**
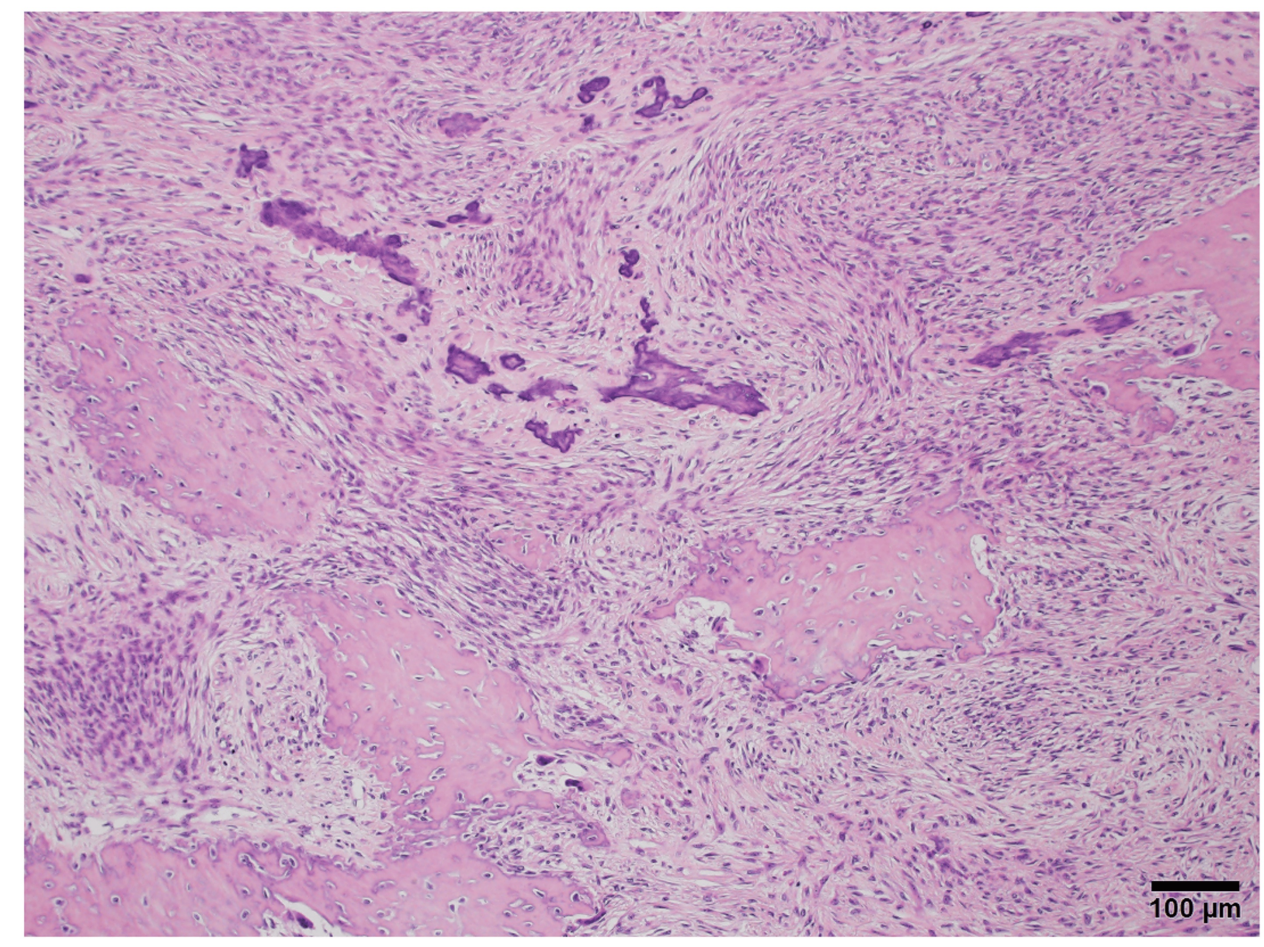
Histopathological findings Hematoxylin and eosin (H&E) Scale bar indicates 100 µm

After securing the nerve stumps within the conduit, the tray was filled with PCBM harvested from the iliac crest and rigidly fixated with titanium screws. Care was taken to avoid compression of the nerve conduit during grafting. The final histopathological examination of the resected specimen confirmed the diagnosis of COF (Figure 3).

**Figure 4:**
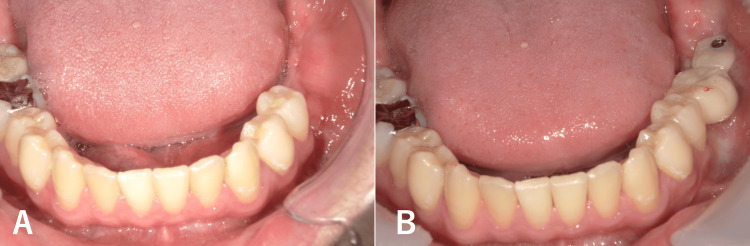
Postoperative intraoral finding A: Before implant placement B: After implant prosthesis delivery

At 18 months postoperatively, a secondary augmentation using iliac cortical bone and PCBM was performed to increase the alveolar bone volume (Figure 4A). 30 months after the initial surgery, two dental implants were placed according to a digital surgical plan, followed by the delivery of a final prosthesis six months later (Figure 4B).

At 18 months postoperatively, a neurosensory assessment using the Semmes-Weinstein monofilament (SW) test and the Numerical Rating Scale (NRS) revealed partial sensory recovery of the lower lip and adjacent skin, with SW values ranging from 3.84 to 3.61 and an NRS score of 8 (Table 1).

**Table 1:** SW test and NRS results (criteria for evaluation） A: Angular branch; B: Inferior labial branch; C: Mental branch Normal: 1.65-2.83; reduced tactile sensation: 3.22-3.61; decreased protective sensation: 3.84-4.31; loss of protective sensation: 4.56-6.65 (Unmeasurable: 6.65） NRS ranges from 0 (complete numbness) to 10 (no numbness). SW: Semmes-Weinstein monofilament; NRS: Numerical Rating Scale

	Time point	Contralateral side	Preoperative	Postoperative	1 month	2 months	4 months	6 months	12 months	＞18 months
SW Test	A	1.65	1.65	6.65	5.46	5.18	4.93	4.74	4.08	3.84
	B	1.65	1.65	6.65	5.46	5.18	4.74	4.74	4.08	3.61
	C	1.65	1.65	6.65	5.46	5.18	5.18	5.18	4.17	3.84
NRS		10	10	0	2	3	4	5	7	8

Postoperative imaging demonstrated stable bone regeneration (Figure 6A) and the formation of a distinct canal-like structure within the reconstructed mandible (Figure 6B).

**Figure 5:**
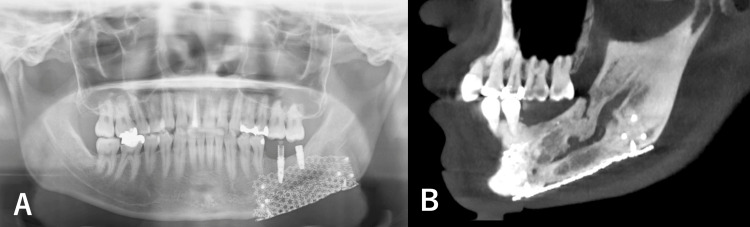
Postoperative radiograph findings A: Panoramic radiograph after the placement of dental implants. B: CBCT image. Postoperative radiographs show satisfactory bone regeneration and formation of a mandibular-like lumen. CBCT: Cone-beam CT

At the 6.5-year follow-up, the patient remained free of recurrence and was highly satisfied with the functional and aesthetic outcomes.

## Discussion

The treatment of this large COF mandated a segmental mandibulectomy due to the lesion’s extent, associated root resorption, and its displacement of the IAN inferiorly, with partial encasement and obscuration of the neurovascular bundle, rendering nerve preservation unfeasible without compromising complete excision. In such cases, radical resection is prioritized to minimize the risk of residual lesion and recurrence [7]. For reconstruction, our rationale was to prioritize a minimally invasive approach appropriate for a young patient with a benign tumor. Therefore, we selected a custom-made titanium mesh tray with a PCBM graft. This technique presents a compelling alternative to vascularized free flaps, circumventing significant donor site morbidity while achieving excellent restoration of the skeletal contour necessary for subsequent prosthetic rehabilitation [2,4,6].

Reconstructing the mandible following resection for tumors or other pathologies presents a formidable clinical challenge, profoundly affecting both function and aesthetics and thus requiring a multidisciplinary approach to achieve optimal outcomes. While vascularized free flaps are considered the gold standard for extensive defects, their associated donor site morbidity and prolonged surgical duration warrant consideration of other options [1,2,4,6]. Particularly for benign tumors in young patients, less invasive and more aesthetic alternatives should be prioritized [4].

In this context, recent studies have reported that mandibular reconstruction using a combination of PCBM grafts with a custom-made titanium mesh tray is an effective alternative that yields favorable outcomes [2,4,6]. This approach was applied in the present case, as it enables the construction of an optimal skeletal framework that addresses both the aesthetic and functional requirements for final prosthetic rehabilitation. A significant and often unavoidable consequence of partial mandibular resection is the severance of the IAN, which can result in permanent sensory loss in the affected area-an issue of significant concern for patient quality of life. Conventionally, nerve reconstruction is performed using autologous nerve grafts, such as the auricular or sural nerve; however, this method carries the inherent risk of creating sensory deficits at the donor site [3-5]. In recent years, nerve guidance conduits have been introduced as a viable alternative to autologous grafts for managing nerve transections or defects. These conduits, typically composed of PGA and filled with collagen, have been shown to be effective for bridging peripheral nerve defects of up to 40 mm in length [4].

The treatment plan in this case was heavily influenced by the patient's expressed desire for an implant-based functional restoration. This necessitated a forward-thinking surgical strategy. To accommodate future implant placement, the nerve conduit's positioning can be strategically adjusted to lie inferior, buccal, or lingual to the original course of the mandibular canal, thereby preserving sufficient vertical bone height for the implants [3,5]. The nerve defect measured precisely 40 mm, the upper limit for which conduit-based regeneration is indicated [4]. Consequently, the nerve conduit was placed in a direct, linear configuration to minimize the gap length and maximize the potential for regeneration. Subsequently, the PCBM graft was carefully applied superior to the conduit to augment the bone volume required for implant placement. Notably, postoperative CT imaging revealed the formation of a distinct, canal-like structure resembling the native mandibular canal, suggesting this was the pathway of the regenerated IAN. In accordance with standard safety protocols for the actual mandibular canal, a minimum distance of 2 mm was maintained from this neo-canal during implant placement [8-10].

Regarding the outcome of nerve reconstruction with a conduit, previous reports have indicated that IAN repair can yield positive sensory recovery in a high percentage of cases [3,11,12]. However, other studies have clearly demonstrated a correlation between increased gap length and poorer clinical outcomes, particularly when using hollow tube conduits [11,13]. In the present case, a 40-mm nerve defect was bridged. At the 12-month postoperative assessment, the NRS score of 8 and the SW test values (ranging from 3.84 to 3.61) suggested that while significant sensory recovery occurred, a full return to normal sensation was not achieved. This limited recovery may be attributed to the fact that the Nerve Bridge® device was used at the absolute upper threshold of its indicated 40-mm range. Performing nerve reconstruction at the maximum indicated distance may have inherently limited the extent of neural regeneration. Given these constraints, the application of nerve guidance conduits in cases involving extensive nerve defects warrants careful consideration and informed patient counseling.

## Conclusions

The present case demonstrates that a holistic, multi-staged approach is critical for achieving successful functional reconstruction in young patients with extensive mandibular defects. Such a comprehensive strategy must consider tumor removal and bony repair, through nerve reconstruction to implant-based occlusal restoration. Our findings reconfirm that a custom-made titanium mesh tray provides an excellent and adaptable framework for anatomical skeletal reconstruction. However, the outcome of the nerve repair offers a valuable lesson regarding the current limitations of synthetic conduits. The partial sensory recovery in this case, where the conduit was utilized at the maximum extent of its indicated 40-mm range, highlights the challenges posed by large nerve defects. This underscores the importance of managing patient expectations and the necessity to define the true limits of immediate nerve reconstruction. Despite this limitation, the procedure successfully prevented permanent anesthesia and resulted in a highly satisfactory functional and aesthetic outcome for the patient, restoring full occlusal function. This patient-centered approach, which prioritizes quality of life, represents a valuable treatment paradigm that should be considered for similar complex cases.
